# Opioid Affinity of Diazacyclic Peptidomimetic Compounds Derived from Reduced Polyamides

**DOI:** 10.3390/ijms26178249

**Published:** 2025-08-25

**Authors:** Prakash Chaudhari, Ashley Bunnell, Manivannan Yegambaram, Colette Dooley, Adel Nefzi

**Affiliations:** 1 Department of Cellular Biology & Pharmacology, Herbert Wertheim College of Medicine, Florida International University, Center for Translational Science, Port Saint Lucie, FL 34987, USA; pchaudha@fiu.edu (P.C.); abunnell@fiu.edu (A.B.); myegamba@fiu.edu (M.Y.); 2 Independent Researcher, Vero Beach, FL 32960, USA; cdooley@tpims.org; 3 Department of Chemistry and Biochemistry, School of Integrated Science and Humanity, College of Arts, Sciences & Education, Florida International University, 11200 S.W. 8th Street, Miami, FL 33199, USA

**Keywords:** peptidomimetics, fused heterocyclic compounds, multifunctional, opioid, peripheral-restriction, analgesia, drug discovery, solid-phase synthesis

## Abstract

Diaza-peptidomimetics are constrained compounds that mimic the biological efficacy of peptides while offering increased stability. We have previously reported the synthesis of bis-cyclic guanidine heterocyclic peptidomimetics as opioid ligands with mixed μ-, κ- and δ-opioid receptor interactions and their potential activity as novel analgesics. Using the same approach, we report here the synthesis of sulfonated and piperazine-tethered bis-cyclic guanidines and their in vitro screening results from radioligand competition binding assays at the μ- (MOR), δ- (DOR), and κ- (KOR) opioid receptors.

## 1. Introduction

The functional differences among the three opioid μ- (MOR), δ- (DOR) and κ- (KOR) receptors and NOP receptors suggest the feasibility of compounds that produce analgesia without the deleterious side effects of opiate analgesic medications [1,2,3,4,5,6,7]. While there have been more than 50 years’ worth of drug discovery efforts to understand the intricacies of these receptors, the need for additional opiate receptor-specific ligands remains [1,3,4,6,8,9,10]. Moreover, while opioids and nonsteroidal anti-inflammatory drugs (NSAIDs) have long been mainstays of pain treatment, their adverse effects and limited efficacy against many types of pain dictate the development of new analgesics. Structurally diverse ligands may result in effective new analgesics and further aid the study of the mechanisms of analgesic efficacy, addiction, and tolerance [6,11]. The development of a new class of potent small-molecule compounds that exhibit decreased side effects would have a significant impact.

The development of opioids with multifunctional, mixed-agonist activity at some or all four of the opioid receptors has produced potent, clinically useful medications [12,13,14,15,16,17,18,19,20]. These results suggest that new opioids capable of multifunctional, opioid agonist activity may generate more effective analgesia. Supporting this, Majumdar et al. reported the synthesis of a dual-acting KOR and Delta opioid receptor (DOR) agonist [20]. The combined activity was thought to contribute to a 15-fold greater antinociceptive potency compared to morphine, but the absence of MOR agonism greatly reduced the effects on respiration and GI transit. The compound did not cause place aversion or preference in mice in a place conditioning assay at doses 3 times the analgesic ED50 value but blocked cocaine-conditioned place preference [20]. The analgesic benefits of mixed-opioid agonist activity have also been shown elsewhere. Schramm and Honda showed that peripheral treatment with DOR agonists enhanced the antinociceptive efficacy of MOR agonists in rats. Additionally, it was found that local intraplantar co-administration of DOR and MOR agonists multiplicatively improved the treatment of pain [21], and loperamide, a peripherally selective opioid agonist with mixed affinity for MOR and DOR, exhibited potent antihyperalgesic and antinociceptive effects in rats [22]. Recently, the Kivell group reported that the mixed Kappa and Delta opioid receptor agonist, MP1104, attenuates chemotherapy-induced neuropathic pain [16]. This data supports the therapeutic development of multifunctional opioid receptor agonists, particularly mixed KOP/DOP agonists, as non-addictive pain medications with reduced tolerance [16]. A potent mix of Mu/Delta/Kappa opioid receptor agonists with reduced likelihood to cause constipation at antinociceptive doses has been described by Spetea [17]. It has been proven that compounds with mixed activity at both Kappa and Mu opioid receptors (e.g., EKC, Mr2033, *cyclo*[Pro-Sar-Phe-D-Phe]) [23] decreased cocaine CPP or self-administration more consistently and with fewer or less severe undesirable side effects than more selective Kappa agonists (e.g., U50,488, spiradoline) [19]. Wilcox and colleagues have further demonstrated that peripherally acting MOR and DOR agonists produce synergistic analgesic activity [24,25]. Although these results support the idea that mixed-action opioid agonists may be of therapeutic value, further development of novel orally active, multifunctional mixed-selectivity agonists is needed to evaluate optimal analgesic utility in treating inflammatory, neuropathic, and cancer-induced pain.

Examples of additional successful compounds with mixed-agonist activity and significantly fewer liabilities of clinical use, such as respiratory distress and addiction, include the following:

- The opioid receptor triple agonist (Delta, Mu, and Kappa receptor) DPI-125. The abuse liability of DPI-125 was evaluated with a self-administration model in rhesus monkeys. The observed agonist potencies of DPI-125 for Delta, Mu, and Kappa opioid receptors were 4.29 ± 0.36, 11.10 ± 3.04, and 16.57 ± 4.14 nmol/L, respectively. DPI-125 exhibited a high respiratory safety profile, clearly related to its high Delta receptor potency. The ratio of the EC_50_ potencies for the Mu and Delta receptors was found to be positively correlated with the respiratory safety ratio. DPI-125 has similar potencies for Mu and Kappa receptors, which is likely the reason for its reduced abuse potential [26].
- The dual opioid/NOP receptor agonist analgesic cebranopadol. The preclinical testing of cebranopadol has characterized it as a dual opioid and NOP receptor agonist that displays antinociceptive and antihyperalgesic action in a variety of acute and chronic pain models in animals. Unlike most current traditional opioids, it is generally more potent against neuropathic than nociceptive pain. Several phase 2 clinical trials have been completed and have moved to phase 3 clinical trials [27].
- The triple agonist DPI-125 is a preclinical-stage opioid drug that acts as a triple agonist at opioid receptors (δ, μ, and κ). It is being developed with the goal of providing effective pain relief with potentially reduced risks of respiratory depression and abuse liability compared to traditional Mu opioid agonists. [26].
- The dual Mu/Delta opioid receptor agonist N-phenethyl substituted 14-O-methylmorphinan-6-ones, which, contrary to N-methylmorphinans, produce effective and potent antinociception without motor impairment in mice [28].
- The dual Mu opioid receptor/nociception–orphanin FQ peptide receptor agonist BPR1M97. It is a potent, rapid analgesic with improved side effects in comparison to morphine. Its novel chemical structure provides novel insights into opioid-managed pain and may be used as a prototype of dual MOP/NOP full agonists [29].
- The dual Mu/Delta opioid agonist RV-Jim-C3, which demonstrated potent efficacious activity in several in vivo pain models, including inflammatory pain, antihyperalgesia, and antiallodynic with no significant motor impairment [30].
- The dual Kappa and Mu opioid receptor agonists. In vivo studies of salvinorin-based compound **10** showed that it produced analgesic activity while avoiding anxiogenic effects in murine models, thus providing further strong evidence for the therapeutic advantages of dual opioid receptor agonists over selective opioid receptor agonists [31].
- The Mu/Delta opioid agonist SRI-22141, which displays enhanced efficacy in neuropathic pain. It displays greatly reduced tolerance and dependence versus morphine. It also has an anti-inflammatory effect in chemotherapy-induced neuropathy. The efficacy and anti-inflammatory effects are mediated by the delta opioid receptor [32].

## 2. Results and Discussion

We previously reported the synthesis of a variety of bis-cyclic guanidine heterocyclic peptidomimetics from reduced tripeptides [33]. In vitro screening with radioligand competition binding assays demonstrated variable affinity for the Mu opioid receptor (MOR), Delta opioid receptor (DOR), and Kappa opioid receptor (KOR) across the series, with compound **1968-22** displaying good affinity for all three receptors with significant antinociception up to 80 min after oral administration (10 mg/kg, p.o.). Compound **1968-22** was detected in the brain 5 min after intravenous administration and was shown to be stable in the blood for at least 30 min. Central administration of **1968-22** did not produce significant respiratory depression, locomotor effects, acute antinociceptive tolerance, or conditioned place preference or aversion. The data suggests that bis-cyclic guanidine heterocyclic peptidomimetics with multifunctional opioid receptor activity may hold potential as new analgesics with fewer liabilities of use.

Extending our earlier studies, synthesizing novel agents from resin-bound tripeptides [34,35,36], we report herein the synthesis and in vitro characterization of opioid affinity of a series of sulfonated and piperazine-tethered bis-cyclic guanidines.

**Scheme 1:**
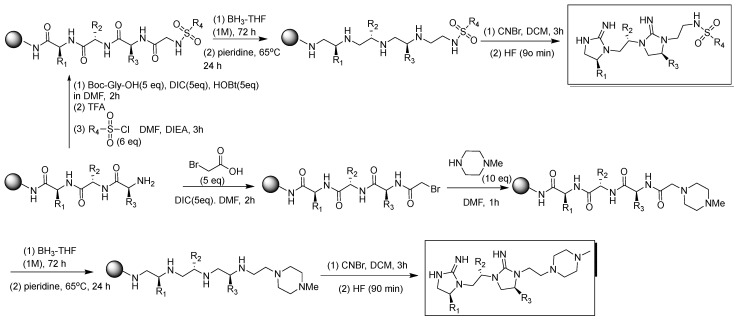
Synthetic strategy for parallel synthesis of sulfonated and piperazine-tethered bis-cyclic guanidines.

The parallel synthesis of all compounds was prepared using our “tea-bag” technology [36,37] following the strategy outlined in Scheme 1. Starting from resin-bound tripeptides, the N-terminal tripeptide was treated with 4-(tert-butyl)benzenesulfonyl chloride in the presence of DIEA in anhydrous DCM. The exhaustive reduction in polyamides was performed using our established BH_3_–THF complex procedure [38,39,40] (Note: the sulfonamide is stable in the reduction reaction conditions [41]). The resulting resin-bound tetraamines were treated with CNBr, and the final compounds were cleaved from the solid support in the presence of anhydrous HF and then extracted with acetic acid and lyophilized. The obtained powder was re-dissolved in a 1:1 mixture of acetonitrile and water, frozen, and subsequently lyophilized. This cycle was repeated twice to obtain solid white powder. All compounds were obtained in crude yields ranging from 72 to 75% (Supplementary Material).

**Figure 1:**
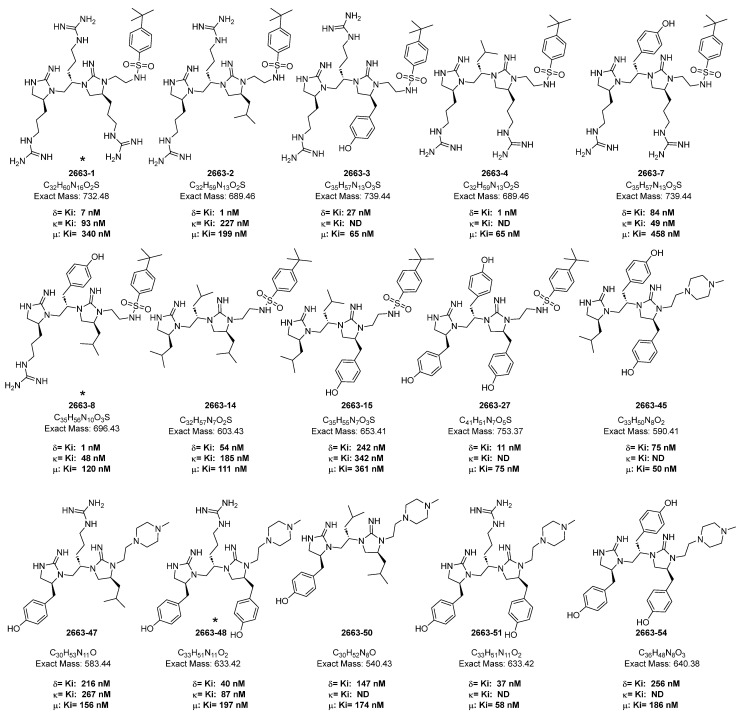
Chemical structures of sulfonated and piperazine-tethered bis-cyclic guanidine heterocyclic peptidomimetics and their affinities for µ- (MOR), δ- (DOR), and κ- (KOR) opioid receptors. * Previously reported compounds.

All synthesized compounds were tested in vitro with radioligand competition binding assays to measure their affinities for MOR, DOR, and KOR, and the inhibition of [3H]DAMGO, [3H]DPDPE, and [3H]U69,593, respectively [38,42,43,44], from their rat brain membrane binding sites. The calculated inhibitory constants (Ki) of the analogues are listed underneath their structures in Figure 1. Most of the compounds displayed good affinity for μ- (MOR), δ- (DOR), and κ- (KOR) opioid receptors.

As we previously reported, compound **2663-48** demonstrated DOR partial agonism and displayed weak KOR partial agonism and modest partial agonism at high (10 μM) concentrations [35]. To investigate the role of each opioid receptor in the antinociceptive effects of compound **2663-48** after i.c.v. administration, in vivo studies were performed in the 55 °C WWTW test using a combination of genetic and pharmacological approaches [35]. The results demonstrated that **2663-48** trends towards a reduction in analgesic response when any of the three opioid receptors were either genetically absent (in the case of MOR and KOR) or pharmacologically blocked (in the case of DOR), indicating that **2663-48** acts as functionally mixed agonists, engaging multiple opioid receptors to mediate their antinociceptive effects. Following peripheral (intraperitoneal, i.p.) administration and pretreatment with naloxone methiodide (NXM), compound **2663-48** was found not to be peripherally selective in its opioid activity [35].

## 3. Methods and Materials

All reagents and solvents were purchased from various commercial sources (Sigma Aldrich (St. Louis, MO, USA), Chemimpex (Wood Dale, IL, USA), VWR (Radnor, PA, USA), Cambridge Isotopes (Tewksbury, MA, USA), etc.) and used without further purification unless otherwise stated. Compounds were prepared on a solid phase using MBHA resin of 1.15 mmole per gram of resin. ^1^H NMR spectra were recorded at 500 MHz and 400 MHz, and ^13^C NMR spectra were recorded at 125 MHz and 100 MHz in deuterated DMSO, and all chemical shifts are reported in δ units relative to TMS. All L-amino acids used were assumed to be enantiomerically pure from the supplier. LC-MS was performed on crude samples dissolved in 50:50 (acetonitrile and water) at a concentration of 1 mg/mL on a Shimadzu LC-MS equipped with a Vydac C18 column with a gradient of 5–95% formic acid (0.1%) in acetonitrile over 7 min, with their UV traces monitored at λ = 214 and 254 nm. HPLC purification was performed on a Phenomenex Luna 150 × 21.2 mm 5 micron column with a flow rate of 12 mL/min.

All compounds were synthesized following the strategy outlined in Scheme 1. The parallel solid-phase synthesis was performed using the “tea-bag” methodology [36,37].

1-(4-(4-(4-hydroxybenzyl)-2-imino-3-(2-(4-methylpiperazin-1-yl)ethyl)imidazolidin-1-yl)-5-(5-(4-hydroxybenzyl)-2-iminoimidazolidin-1-yl)pentyl)guanidine (2663-48): ^1^H NMR (400 MHz, DMSO) δ 8.59 (s, 1H), 8.28 (s, 1H), 8.08 (s, 1H), 7.10 (d, *J* = 8 Hz, 4H), 6.72 (t, *J* = 8 Hz, 5H), 5H), 4.41 (m, 2H), 4.14 (m, 2H), 3.46–3.25 (m, 9H), 3.15 (d, *J* = 13.5 Hz, 7H), 3.02 (d, *J* = 13.3 Hz, 3H), 2.92 (d, *J* = 14.2 Hz, 3H), 2.83 (s, 1H), 2.77 (s, 3H), 2.71–2.55 (m, 4H), 2.48–2.37 (m, 2H), 1.51 (s, 3H), 1.45 (s, 2H), 1.26 (dd, *J* = 13.1, 6.5 Hz, 1H). ^13^C NMR (125 MHz, DMSO-*d*_6_) 159.4, 159.1, 158.8, 158.5,158.0, 157.5, 156.8, 156.7, 134.8, 131.0, 130.7, 126.5, 126.0,118.9, 115.9, 115.8, 115.7, 58.3, 58.2, 54.0, 53.8, 52.9, 52.1, 49.8, 46.4, 45.4, 44.7, 42.7, 42.4, 38.0, 35.6, 25.7, 25.3, 18.5, 17.0 MS (ESI) *m*/*z* [M + H]^+^: 634.85.

4-(tert-butyl)-N-(2-((S)-3-((S)-5-guanidino-1-((S)-5-(3-guanidinopropyl)-2 iminoimidazolidin1-yl)pentan-2-yl)-5-(3-guanidinopropyl)-2-iminoimidazolidin-1-yl)ethyl)benzenesulfonamide (2663-1): ^1^H NMR (400 MHz, DMSO) δ 8.50 (1H), 8.40 (1H), 8.25 (1H), 8.00–7.96 (m, 4H), 7.76 (d, *J* = 8 Hz, 2H), 7.64 (d, *J* = 8 Hz, 2H), 7.41–7.00 (m, 6H), 5.81 (d, 1H), 3.16–3.14 (m, 14H), 2.76 (m, 2H), 2.55 (m, 3H), 1.58–1.42 (m, 16H), 1.31 (s, 3H). ^13^C NMR (125 MHz, DMSO-*d*_6_) δ 157.5, 156.04, 137.81, 130.5, 130.0, 127.1, 126.9, 126.6, 118.9, 116.0, 115.4, 53.9, 42.6, 35.31, 31.3, 18.5, 17.2, 12.7. MS (ESI) *m*/*z* [M + H]^+^: 733.48.

4-(tert-butyl)-N-(2-((S)-3-((S)-1-((S)-5-(3-guanidinopropyl)-2-iminoimidazolidin-1-yl)-3-(4-hydroxyphenyl)propan-2-yl)-2-imino-5-isobutylimidazolidin-1-yl)ethyl)benzenesulfonamide (2663-8): ^1^H NMR (400 MHz, DMSO) δ 9.39 (s, 1H), 8.43 (s, 1H), 8.34 (s, 1H), 8.26 (s, 1H), 8.03 (s, 1H), 7.74 (d, *J* = 8 Hz, 2H), 7.64 (d, *J* = 8 Hz, 2H), 7.18 (d, *J* = 8 Hz, 2H), 6.72 (d, *J* = 8 Hz, 2H) 4.12 (s, 1H), 3.97–3.94 (m, 2H), 3.73 (t, *J* = 12 Hz, 1H), 3.19–3.10 (m, 4H), 2.87–2.72 (m, 4H), 2.55 (m, 10H), 1.57 (m, 2H), 1.47 (m, 2H), 1.31 (s, 9H), 0.87 (dd, *J* = 16 and 8 Hz, 6H). ^13^C NMR (125 MHz, DMSO-*d*_6_) δ 159.15, 158.84, 158.08, 157.48, 156.72, 156.09, 137.71, 130.48, 126.86, 126.75, 126.64, 119.11, 116.13, 115.76, 55.52, 53.96, 40.93, 40.64, 40.43, 40.22, 40.01, 39.81, 39.60, 39.39, 35.32, 31.26, 24.42, 23.93, 21.67, 18.49, 17.19. MS (ESI) *m*/*z* [M + H]^+^: 696.43.

## 4. Conclusions

In conclusion, continuing with our efforts to generate new opioids from modified short peptides [34], we synthesized a series of sulfonated and piperazine-tethered bis-cyclic guanidines and determined their affinities against μ (MOR), δ (DOR), and κ (KOR) opioid receptors. Future efforts will be focused on in vivo validation of the compounds with multifunctional, mixed-agonist activity at some or all four of the opioid receptors to continue with our efforts toward the identification of potent, clinically useful analgesics.

## Data Availability

Data is contained within the article or Supplementary Material.

## Supplementary Materials

The following supporting information can be downloaded at https://www.mdpi.com/article/10.3390/ijms26178249/s1.
