# Supplementary material for: Opioid Affinity of Diazacyclic Peptidomimetic Compounds Derived from Reduced Polyamides
Source: Int J Mol Sci. 2025 Aug 25;26(17):8249. doi: 10.3390/ijms26178249 (PMC12428765; doi:10.3390/ijms26178249)
Supplement: Supplementary file 1 [file ijms-26-08249-s001.zip › ijms-3781162-supplementary.pdf]

Opioid Affinity of Diazacyclic Peptidomimetic Compounds Derived from Reduced Polyamides

Prakash Chaudhari [1], Ashley Bunnell [1], Manivannan Yegambaram[1], Colette Dooley [2] and Adel Nefzi [1,3]*.

[1] Department of Cellular Biology & Pharmacology, Herbert Wertheim College of Medicine, Florida International University, Center for Translational Science, Port Saint Lucie, Florida 34987.

[2] Independent Researcher, Vero Beach, Florida, United States

[3] Department of Chemistry and Biochemistry, the School of Integrated Science and Humanity, College of Arts, Sciences & Education. Florida International University, 11200 S.W. 8th Street, Miami, FL 33199; anefzi@fiu.edu

MS spectra of all compounds.

NMR spectra of representative compounds: 2663-1, -8 and -48.

HRMS data of representative compounds.

2663-1

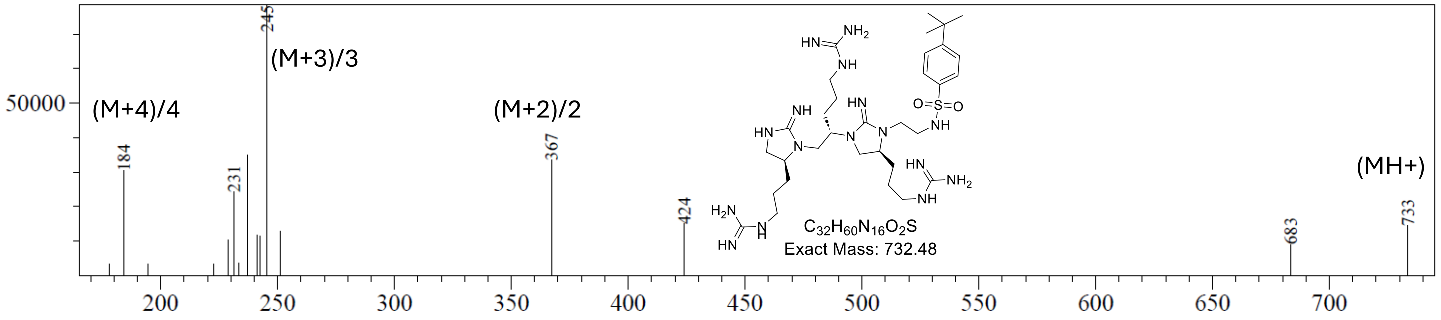

2663-2

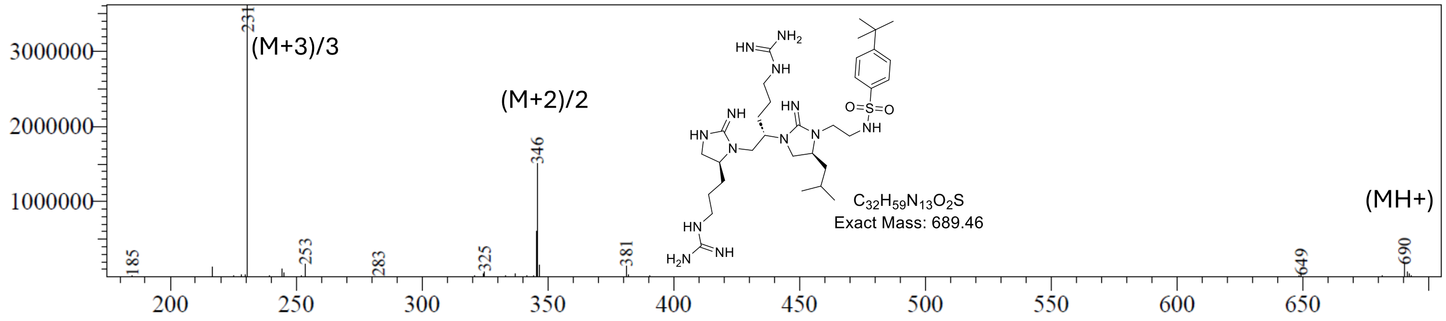

2663-3

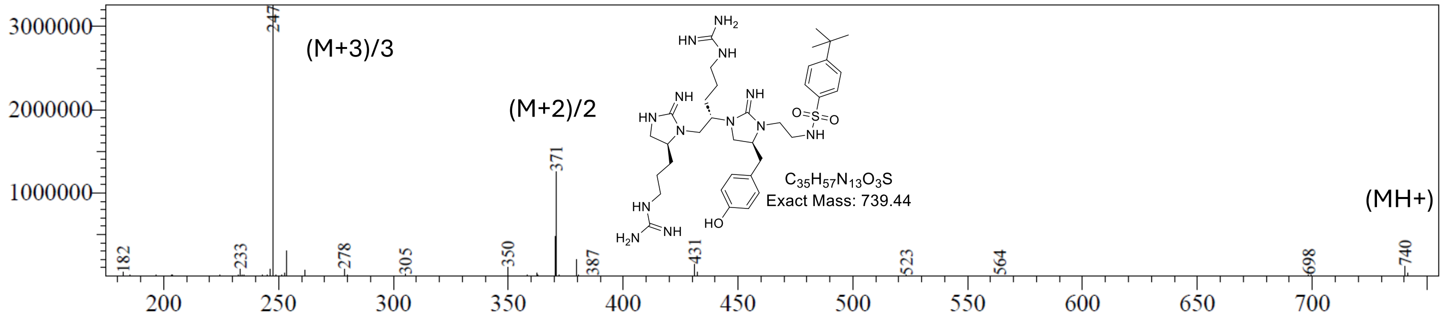

2663-4

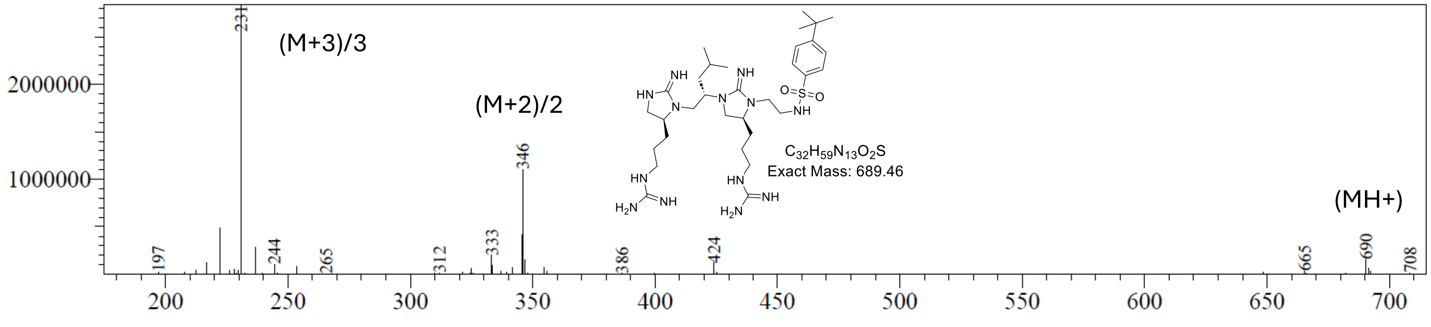

2663-7

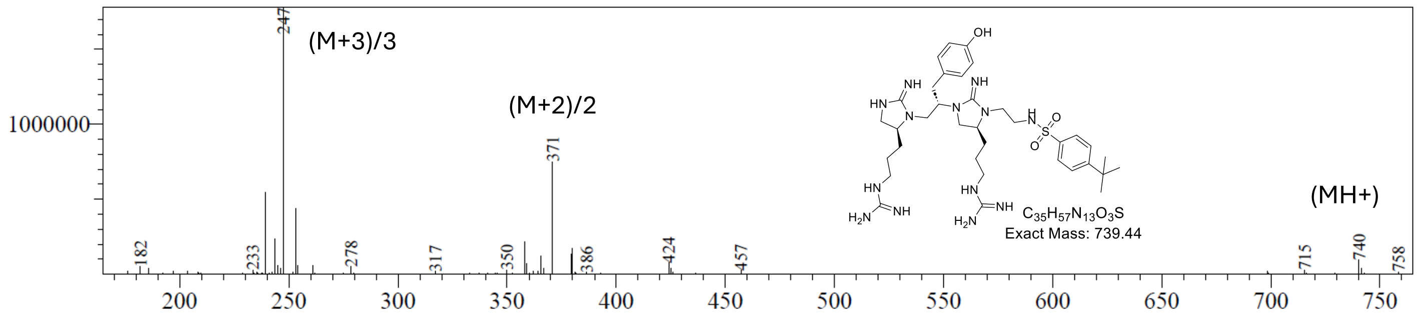

2663-8

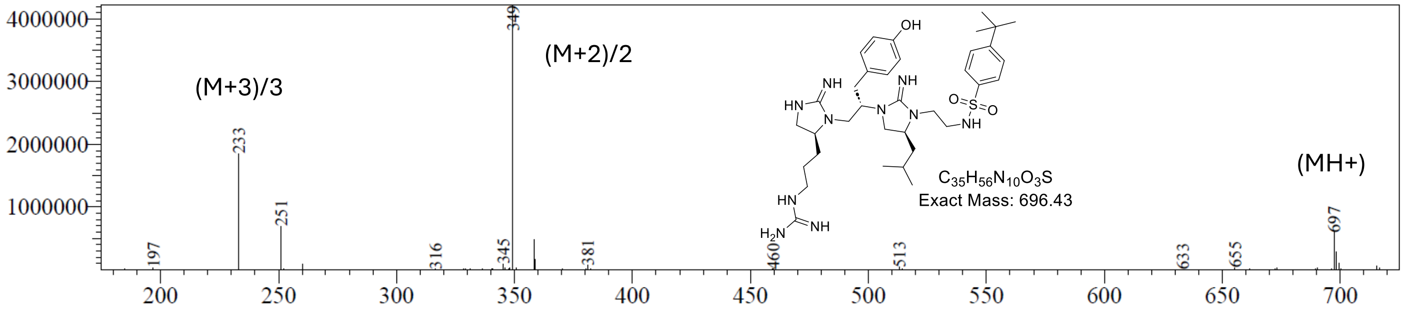

2663-14

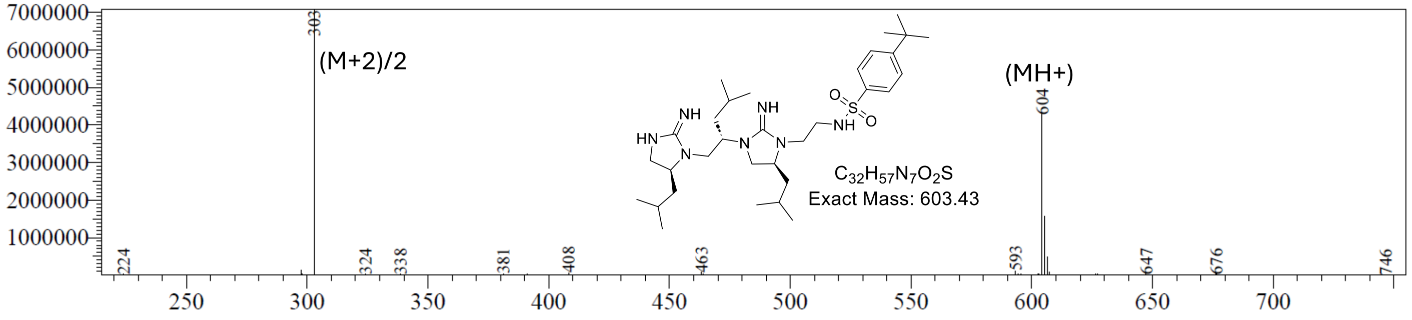

2663-15

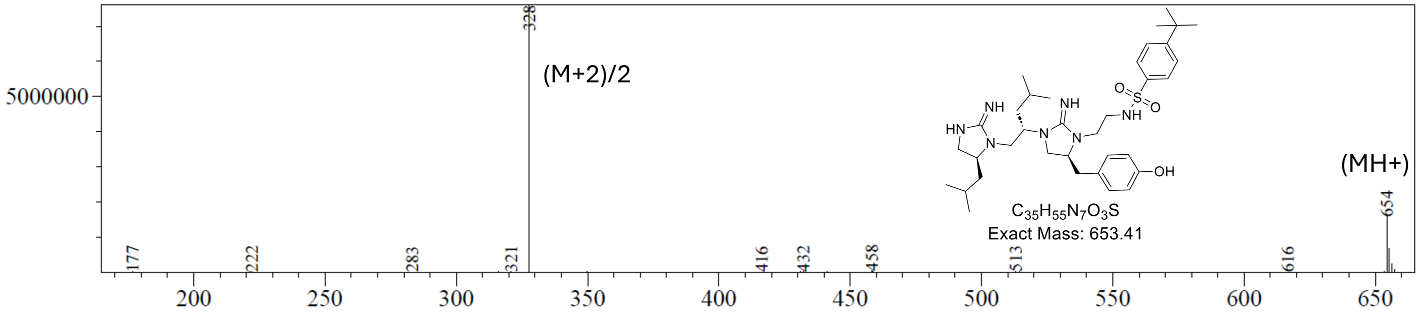

2663-27

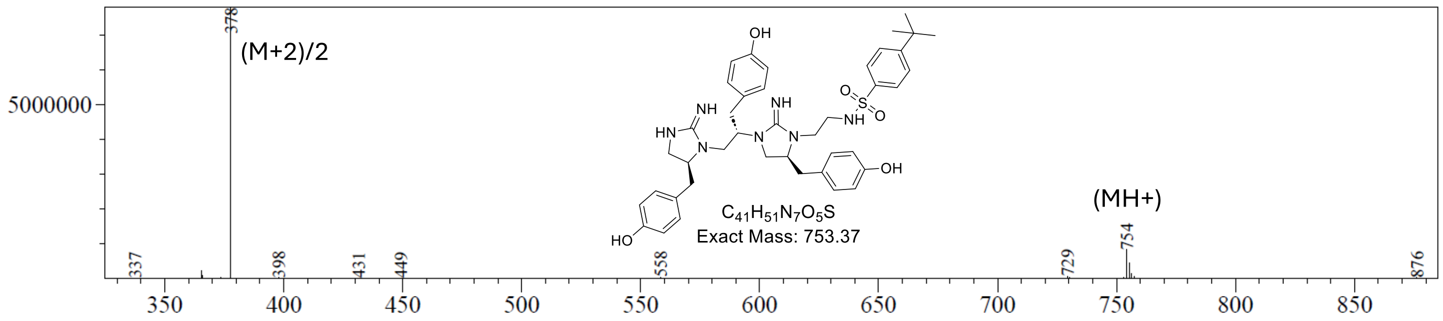

3663-45

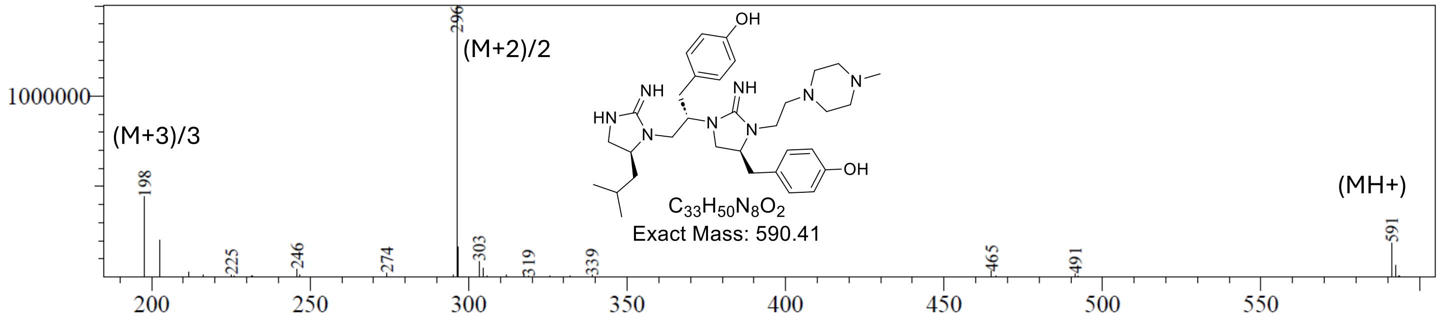

2663-47

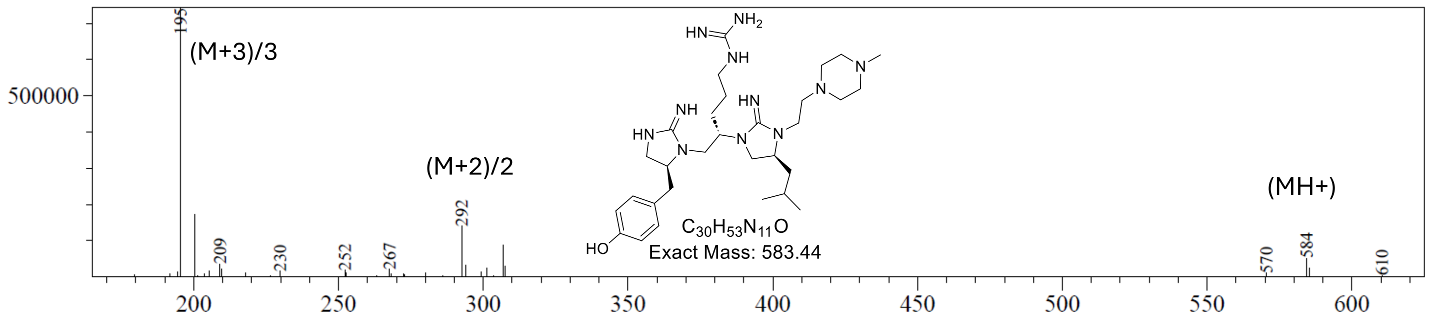

2663-48

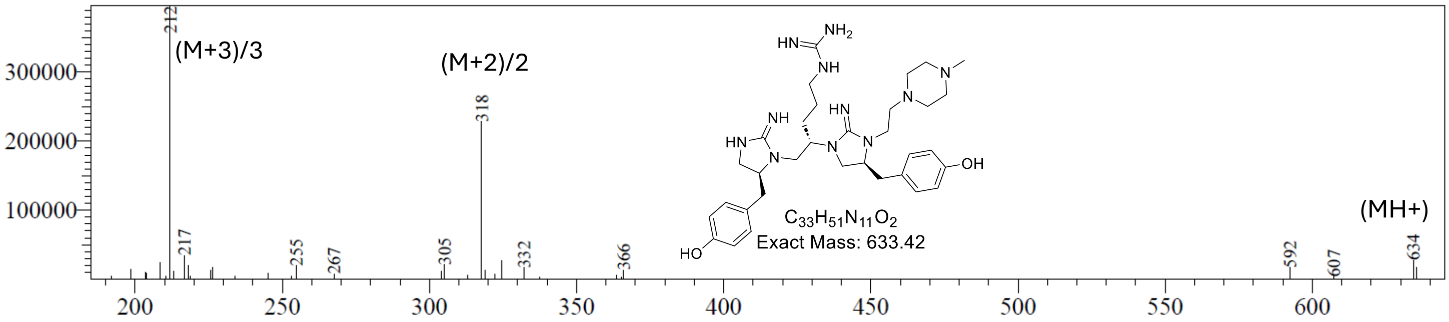

2663-50

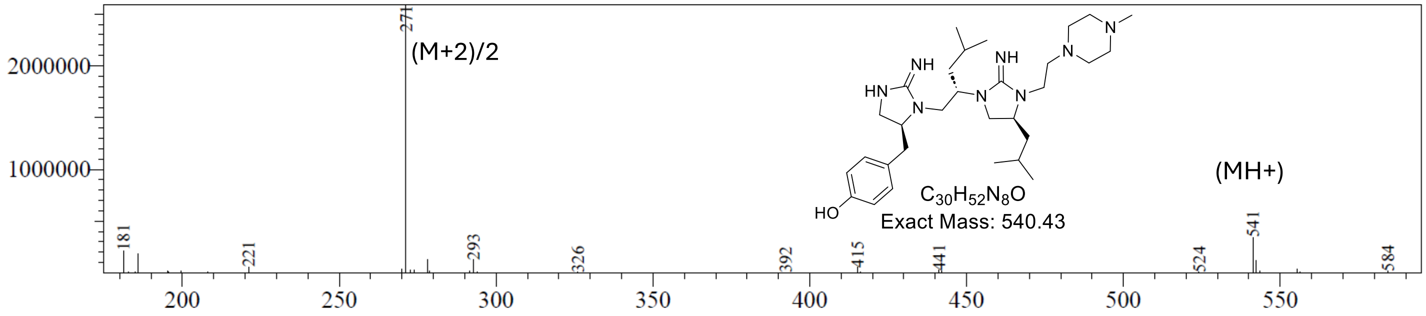

2663-51

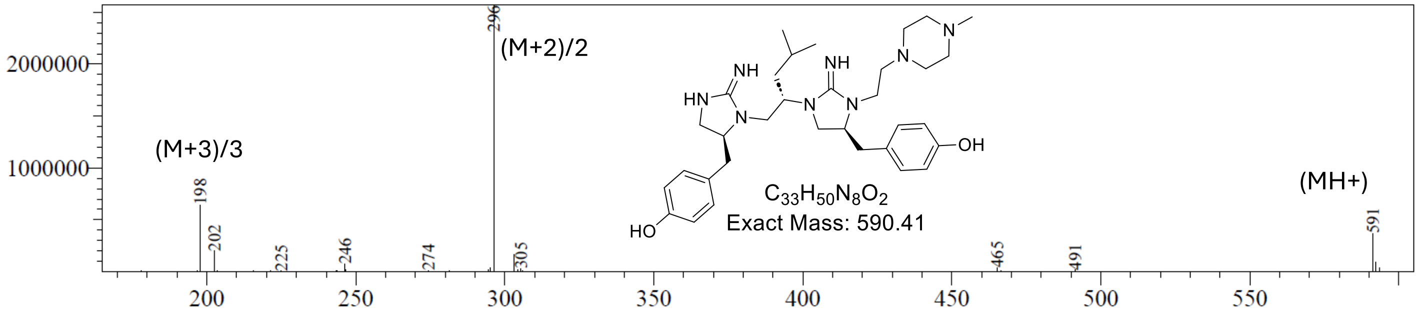

2663-54

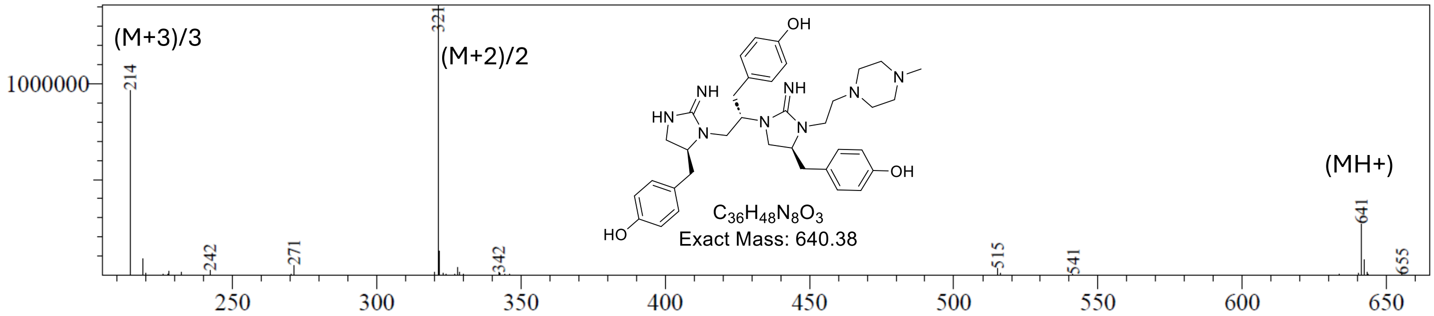

HRMS data

2663-1

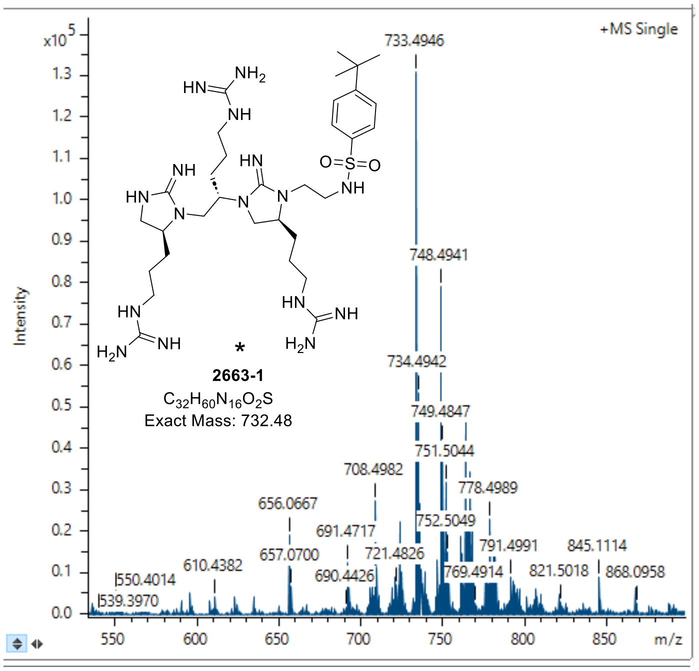

2663-3

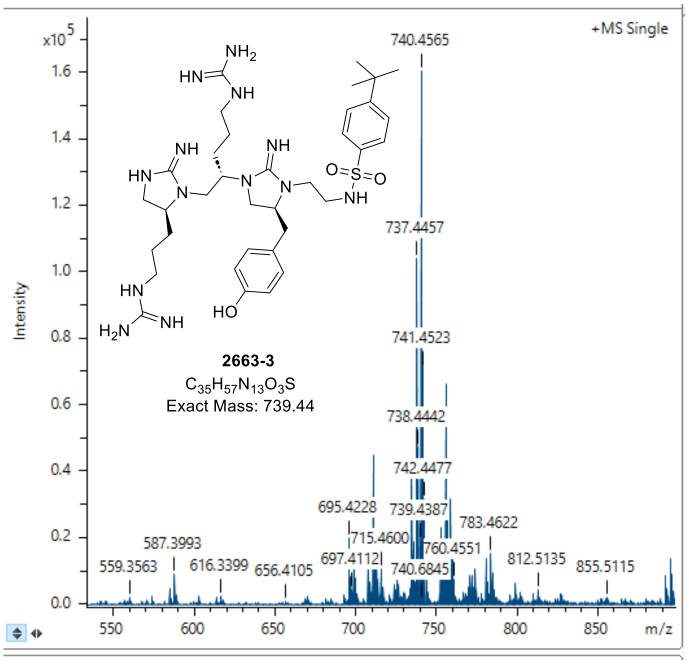

2663-7

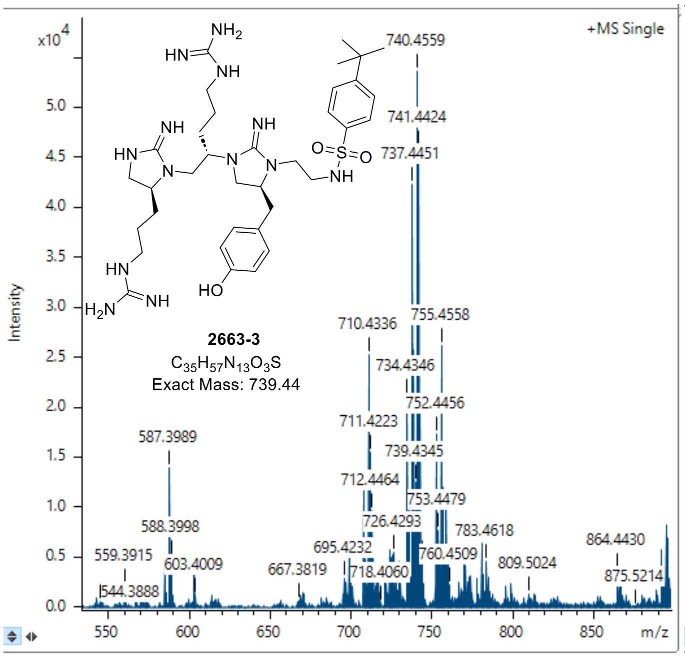

2663-8

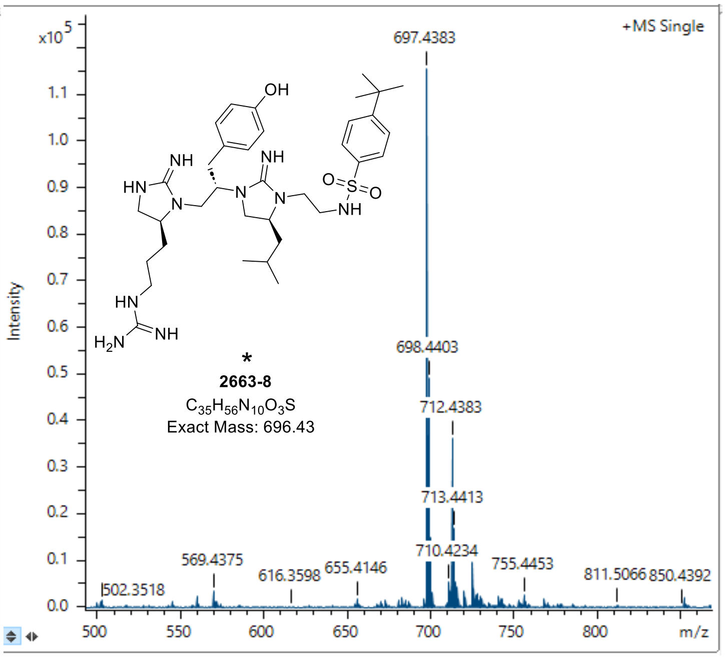

2663-27

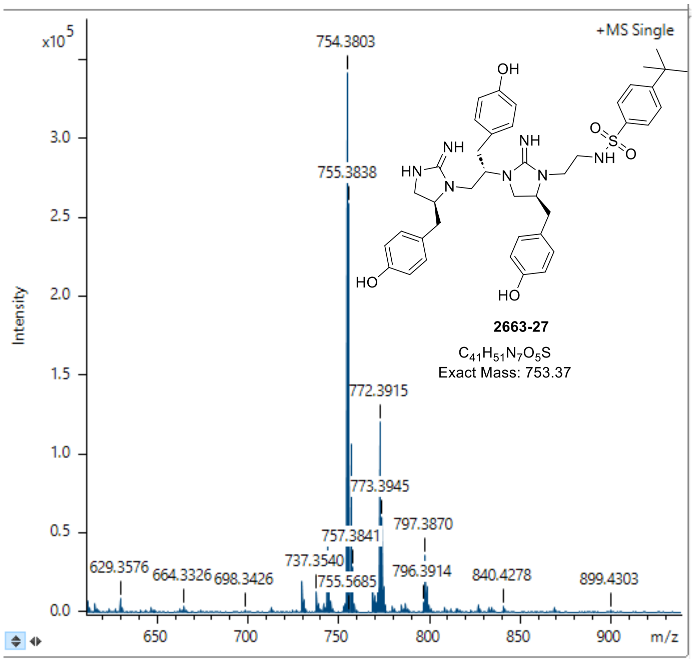

2663-47

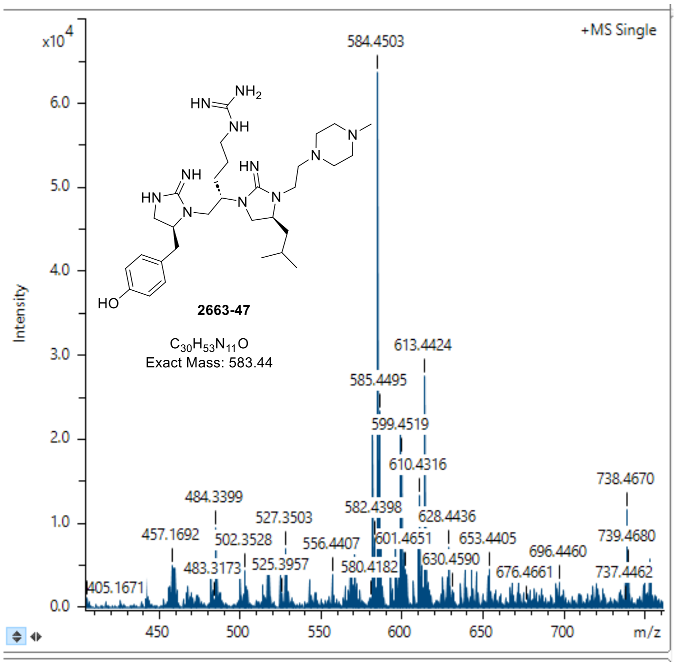

2663-54

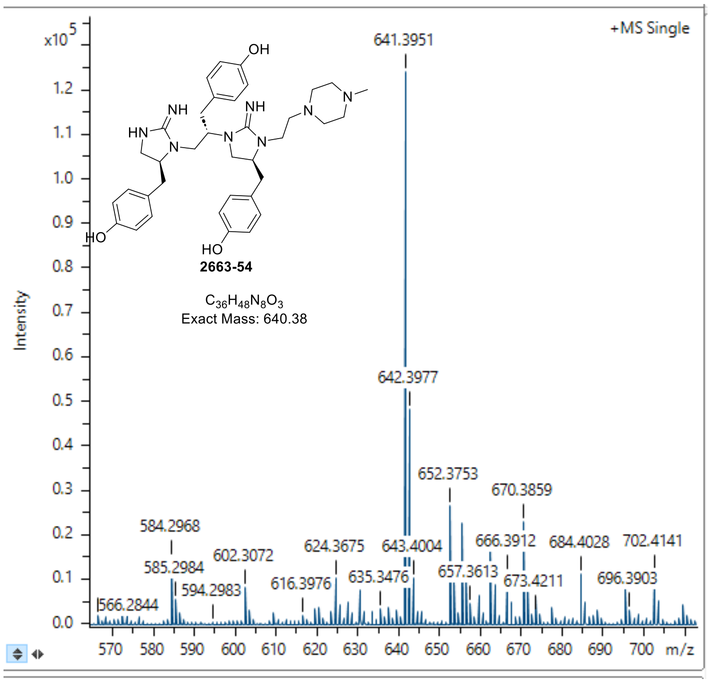

NMR data

Spectral data of 2663-1:

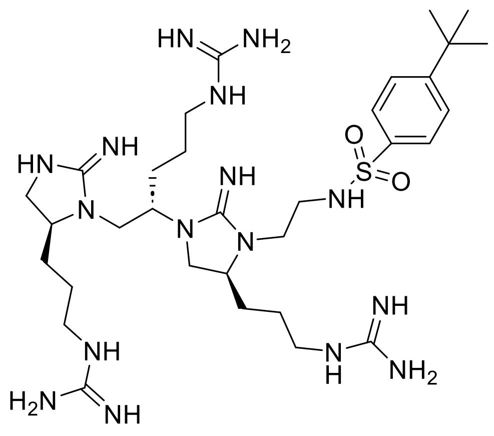

2663-1

4-(tert-butyl)-N-(2-((S)-3-((S)-5-guanidino-1-((S)-5-(3-guanidinopropyl)-2 iminoimidazolidin1-yl)pentan-2-yl)-5-(3-guanidinopropyl)-2-iminoimidazolidin-1-yl)ethyl)benzenesulfonamide:

^{1}H NMR (400 MHz, DMSO) δ 8.50 (1H), 8.40 (1H), 8.25 (1H), 8.00- 7.96 (m, 4H), 7.76 (d, *J* = 8 Hz, 2H), 7.64 (d, *J* = 8 Hz, 2H), 7.41- 7.00 (m, 6H), 5.81(d, 1H), 3.16- 3.14 (m, 14H), 2.76 (m, 2H), 2.55 (m, 3H), 1.58- 1.42 (m, 16H), 1.31 (s, 3H). ^{13}C NMR (125 MHz, DMSO-d_6) δ 157.5, 156.04, 137.81, 130.5, 130.0, 127.1, 126.9, 126.6, 118.9, 116.0, 115.4, 53.9, 42.6, 35.31, 31.3, 18.5, 17.2, 12.7. MS (ESI) m/z [M + H] +: 733.48

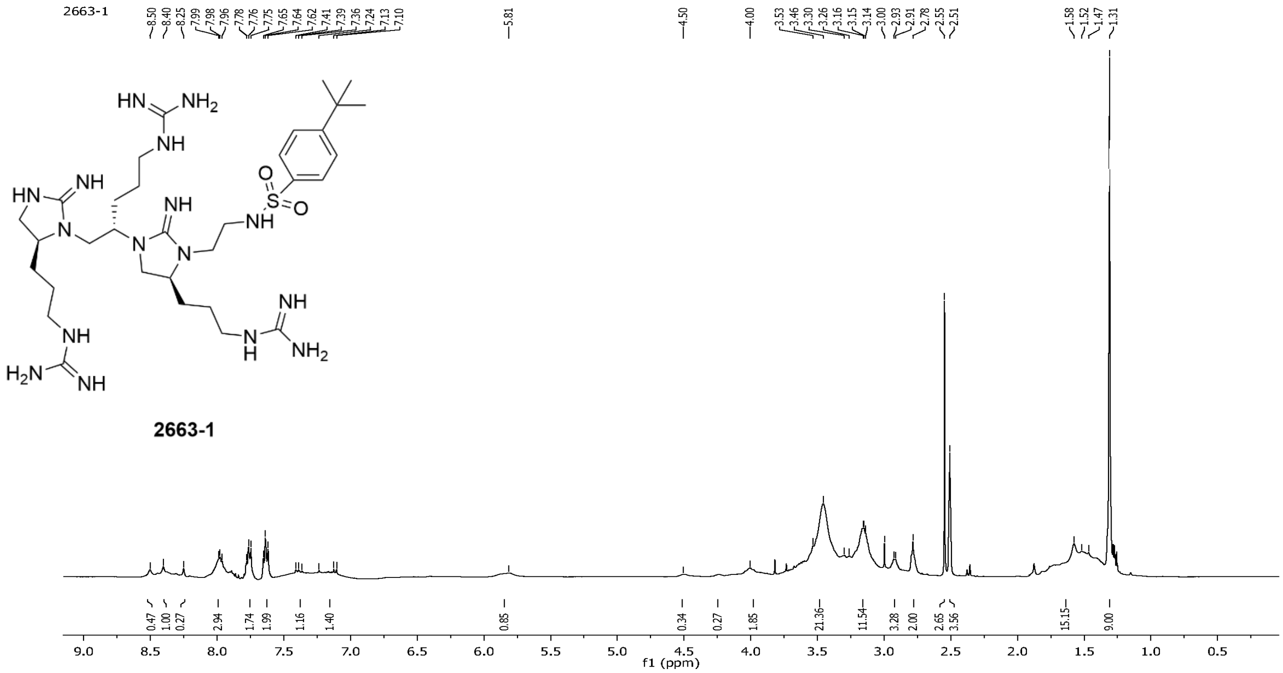

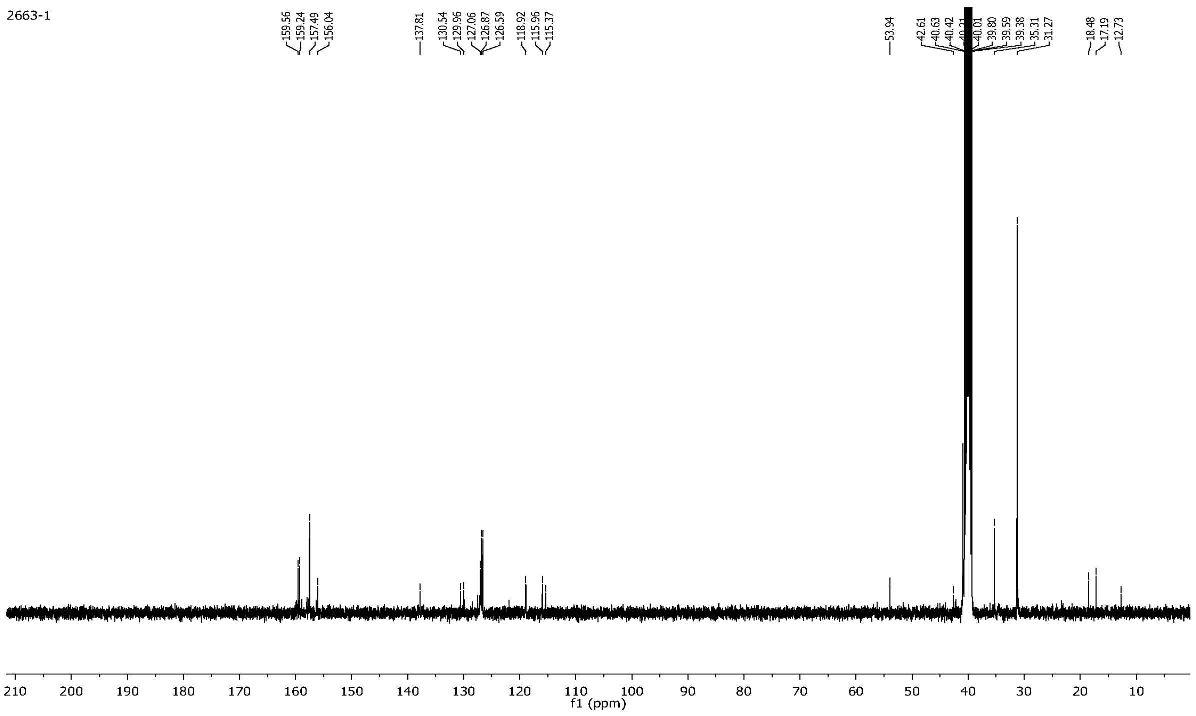

Spectral data of 2663-8:

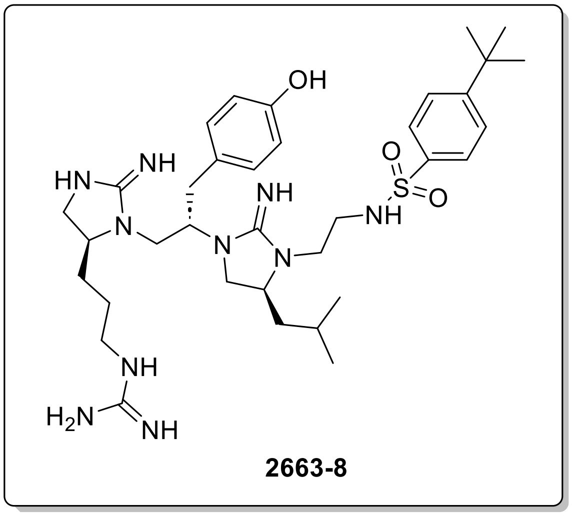

4-(tert-butyl)-N-(2-((S)-3-((S)-1-((S)-5-(3-guanidinopropyl)-2-iminoimidazolidin-1-yl)-3-(4-hydroxyphenyl)propan-2-yl)-2-imino-5-isobutylimidazolidin-1-yl)ethyl)benzenesulfonamide: ^{1}H NMR (400 MHz, DMSO) δ 9.39 (s, 1H), 8.43 (s, 1H), , 8.34 (s, 1H), 8.26 (s, 1H), 8.03 (s, 1H), 7.74 (d, *J* = 8 Hz, 2H), 7.64 (d, *J* = 8 Hz, 2H), 7.18 (d, *J* = 8 Hz, 2H), 6.72 (d, *J* = 8 Hz, 2H) 4.12 (s, 1H), 3.97- 3.94 (m, 2H), 3.73 (t, *J* = 12 Hz, 1H), 3.19- 3.10 (m, 4H), 2.87- 2.72 (m, 4H), 2.55 (m, 10H), 1.57 (m, 2H), 1.47 (m, 2H), 1.31 (s, 9H), 0.87 (dd, *J*= 16 and 8 Hz, 6H). ^{13}C NMR (125 MHz, DMSO-d_6) δ 159.15, 158.84, 158.08, 157.48, 156.72, 156.09, 137.71, 130.48, 126.86, 126.75, 126.64, 119.11, 116.13, 115.76, 55.52, 53.96, 40.93, 40.64, 40.43, 40.22, 40.01, 39.81, 39.60, 39.39, 35.32, 31.26, 24.42, 23.93, 21.67, 18.49, 17.19. MS (ESI) m/z [M + H] +: 696.43

2663-8

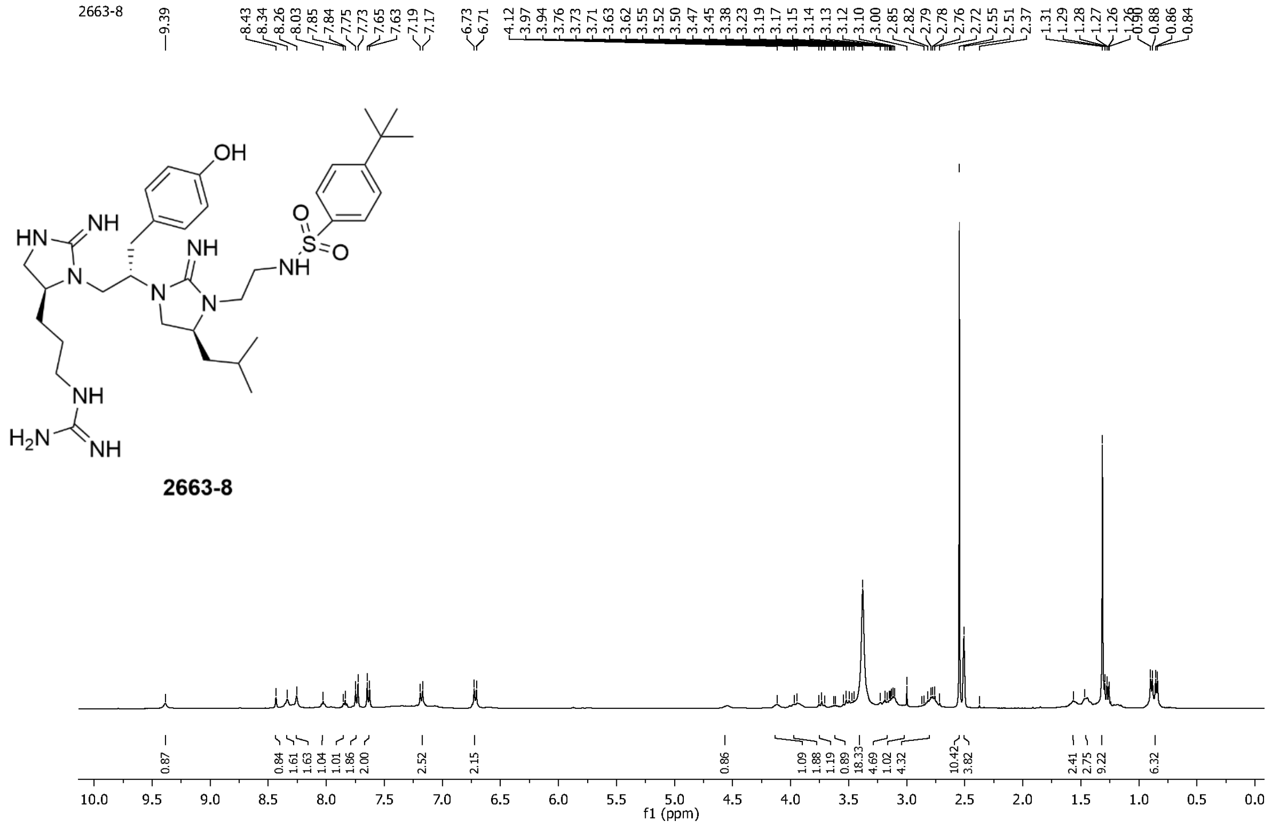

2663-8-13C

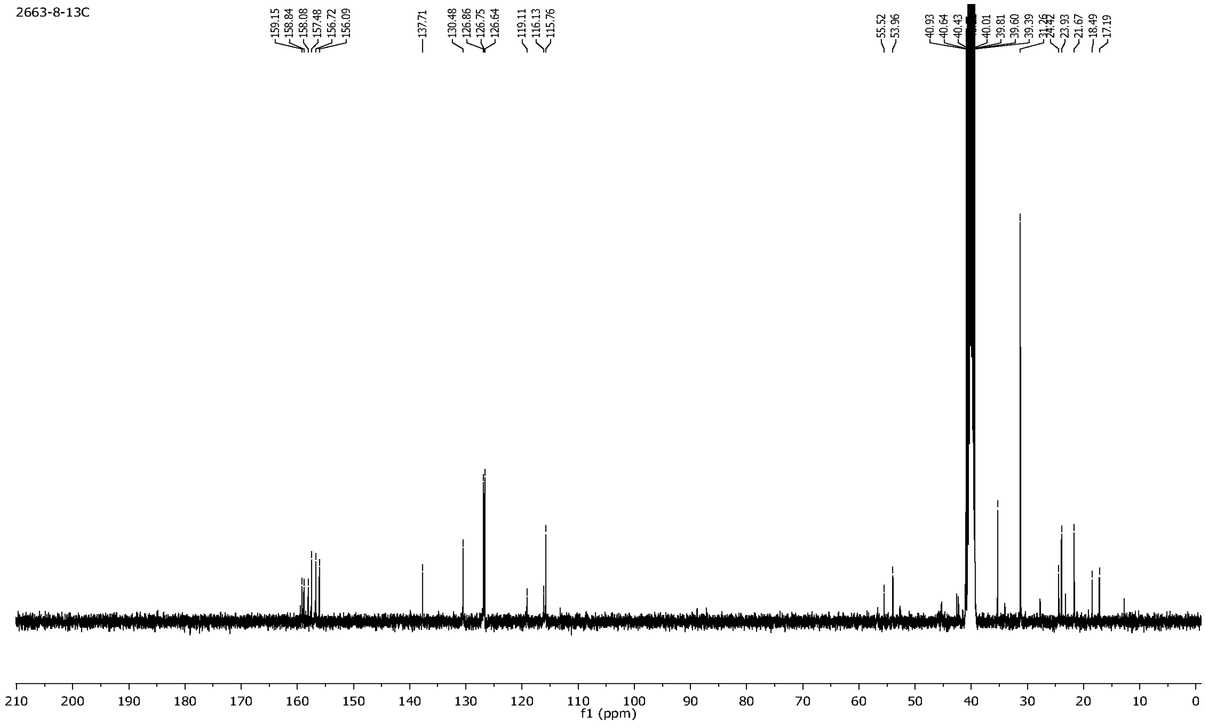

Spectral data of 2663-48:

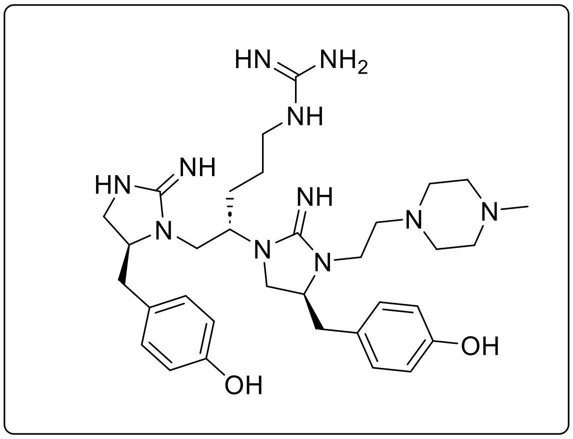

1-(4-(4-(4-hydroxybenzyl)-2-imino-3-(2-(4-methylpiperazin-1-yl)ethyl)imidazolidin-1-yl)-5-(5-(4-hydroxybenzyl)-2-iminoimidazolidin-1-yl)pentyl)guanidine: ^{1}H NMR (400 MHz, DMSO) δ 8.59 (s, 1H), 8.28 (s, 1H), 8.08 (s, 1H), 7.10 (d, J = 8 Hz, 4H), 6.72 (t, J = 8 Hz, 5H) , 5H), 4.41 (m, 2H), 4.14 (m, 2H), 3.46 – 3.25 (m, 9H), 3.15 (d, J = 13.5 Hz, 7H), 3.02 (d, J = 13.3 Hz, 3H), 2.92 (d, J = 14.2 Hz, 3H), 2.83 (s, 1H), 2.77 (s, 3H), 2.71 – 2.55 (m, 4H), 2.48 – 2.37 (m, 2H), 1.51 (s, 3H), 1.45 (s, 2H), 1.26 (dd, J = 13.1, 6.5 Hz, 1H). ^{13}C NMR (125 MHz, DMSO-d_6) 159.4, 159.1, 158.8, 158.5,158.0, 157.5, 156.8, 156.7, 134.8, 131.0, 130.7, 126.5, 126.0,118.9, 115.9, 115.8, 115.7, 58.3, 58.2, 54.0, 53.8, 52.9, 52.1, 49.8, 46.4, 45.4, 44.7, 42.7, 42.4, 38.0, 35.6, 25.7, 25.3, 18.5, 17.0 MS (ESI) m/z [M + H] +: 634.85

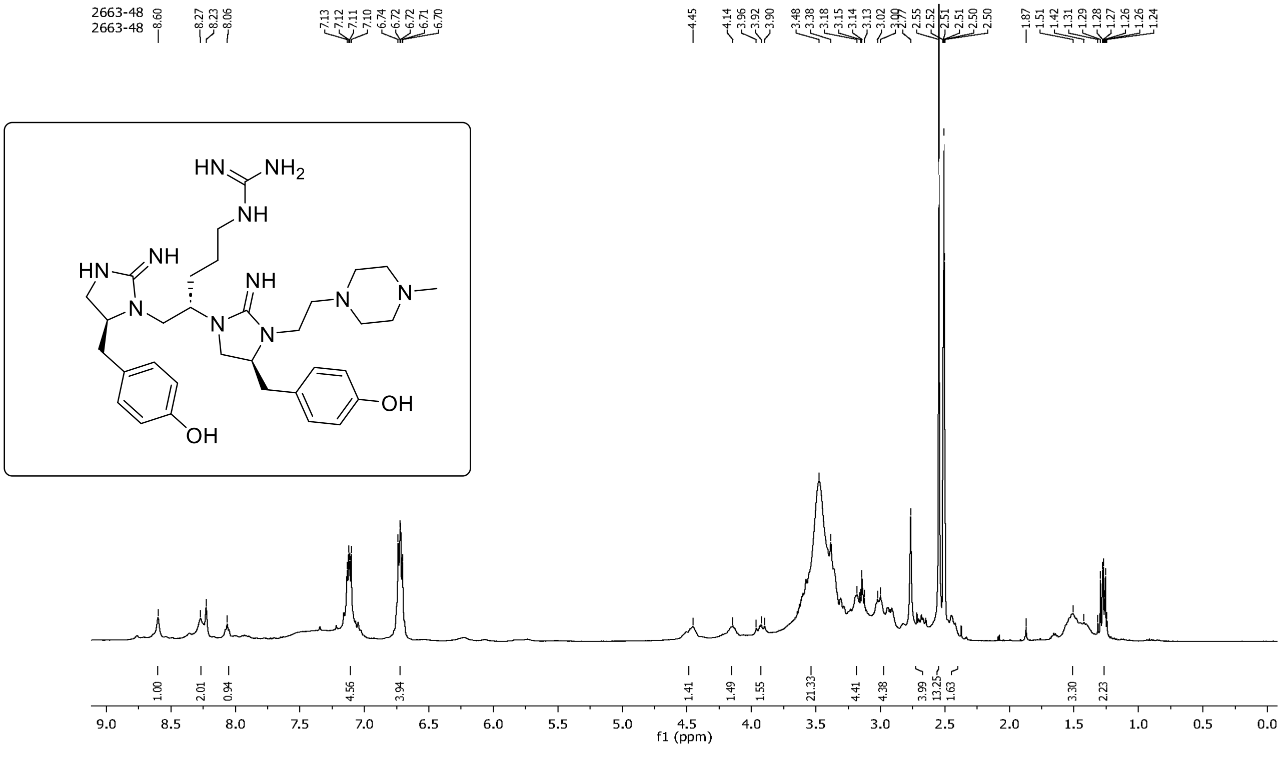

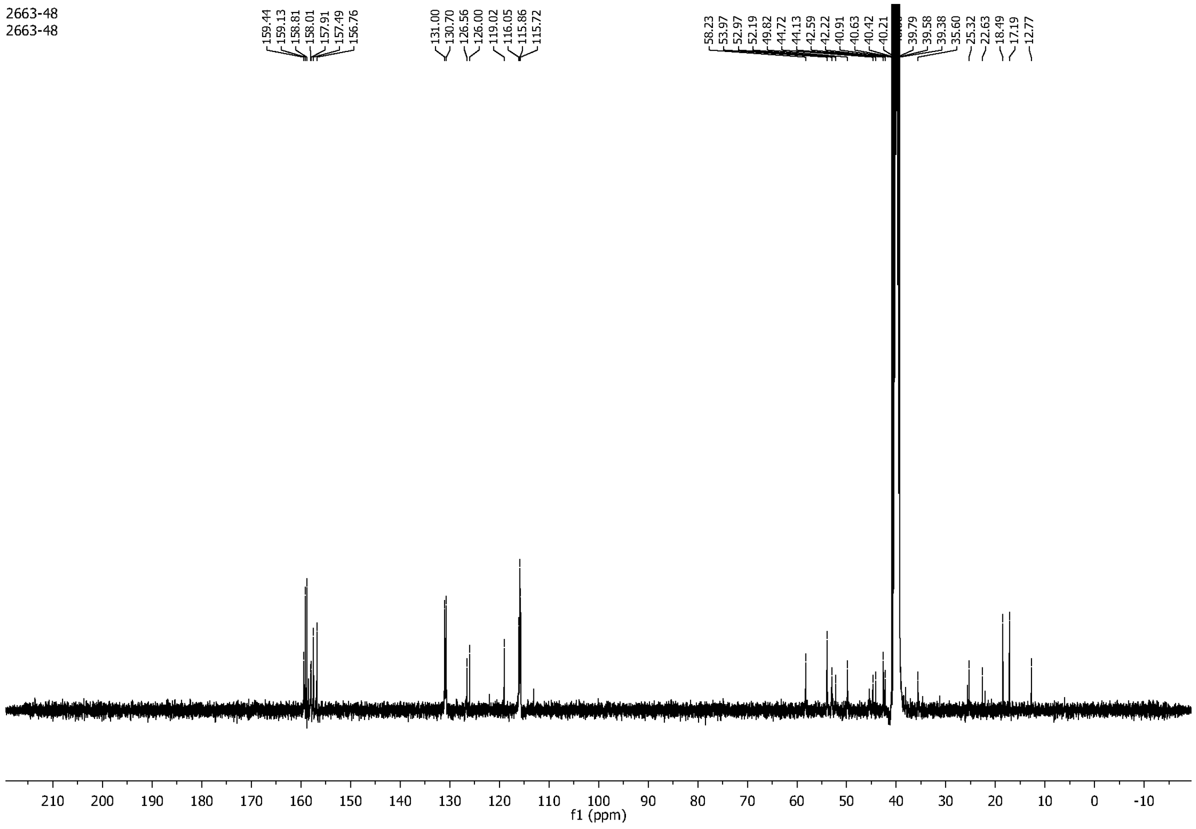

Spectral data of 2663-7:

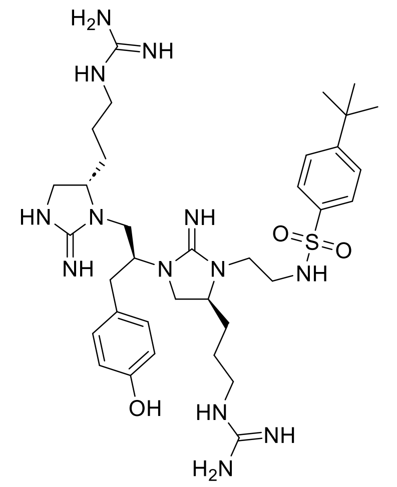

2663-7

4-(tert-butyl)-N-(2-((S)-5-(3-guanidinopropyl)-3-((S)-1-((S)-5-(3-guanidinopropyl)-2-iminoimidazolidin-1-yl)-3-(4-hydroxyphenyl)propan-2-yl)-2-iminoimidazolidin-1-yl)ethyl)benzenesulfonamide: 1H NMR (400 MHz, DMSO) δ 8.37 (d, *J* = 8 Hz, 4H), 8.30 (s, 1H), 8.02 (d, *J* = 8 Hz, 1H), 8.00 (m, 1H), 7.86- 7.83 (m, 1H), 7.78- 7.73 (m, 2H), 7.64- 7.62 (m, 2H), 6.73 (d, *J* = 8 Hz, 2H), 4.14 (m, 1H), 4.07- 4.00 (m, 1H), 3.87- 3.72 (m, 8H), 3.52 (m, 2H), 3.35 (m, 2H), 3.19- 3.17 (m, 7H), 2.81- 2.75 (m, 4H), 1.70 (m, 1H), 1.56- 1.36 (m, 3H), 1.30 (s, 3H); ^{13}C NMR (125 MHz, DMSO) δ 172.4, 159.69, 159.37, 157.51, 156.72, 130.49, 126.81, 118.75, 115.79, 56.56, 40.27, 40.05, 39.96, 39.75, 39.54, 35.29, 31.24. MS (ESI) m/z [M + H] +: 739.44

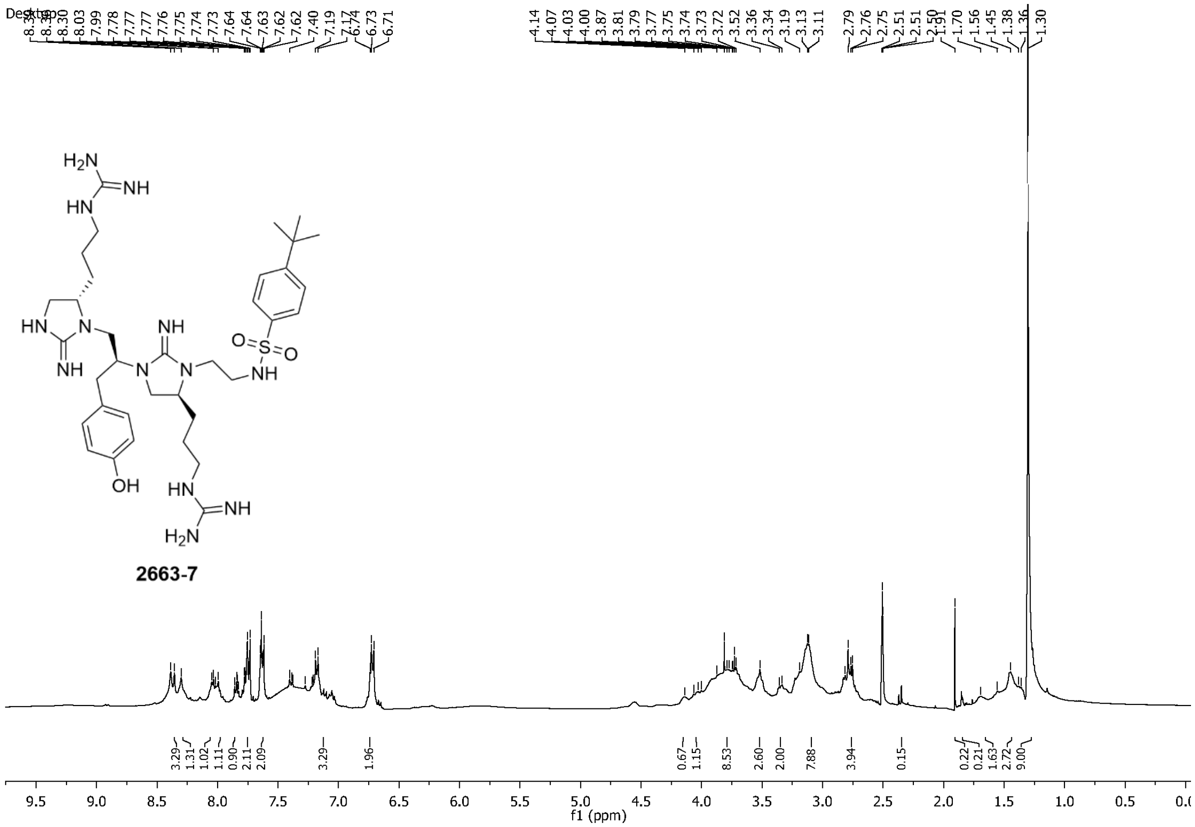
Desktop
8.30
8.03
7.99
7.78
7.77
7.77
7.76
7.75
7.74
7.73
7.64
7.63
7.62
7.62
7.40
7.19
7.17
6.74
6.73
6.71
4.14
4.07
4.03
4.00
3.87
3.81
3.79
3.77
3.75
3.74
3.73
3.72
3.52
3.36
3.34
3.19
3.13
3.11
2.79
2.76
2.75
2.51
2.51
2.50
1.91
1.70
1.56
1.45
1.38
1.36
1.30
2663-7
3.29
1.31
1.02
1.11
0.90
2.11
2.09
3.29
1.96
0.67
1.15
8.53
2.60
2.00
7.88
3.94
0.15
0.22
0.21
1.63
2.72
9.00
f1 (ppm)

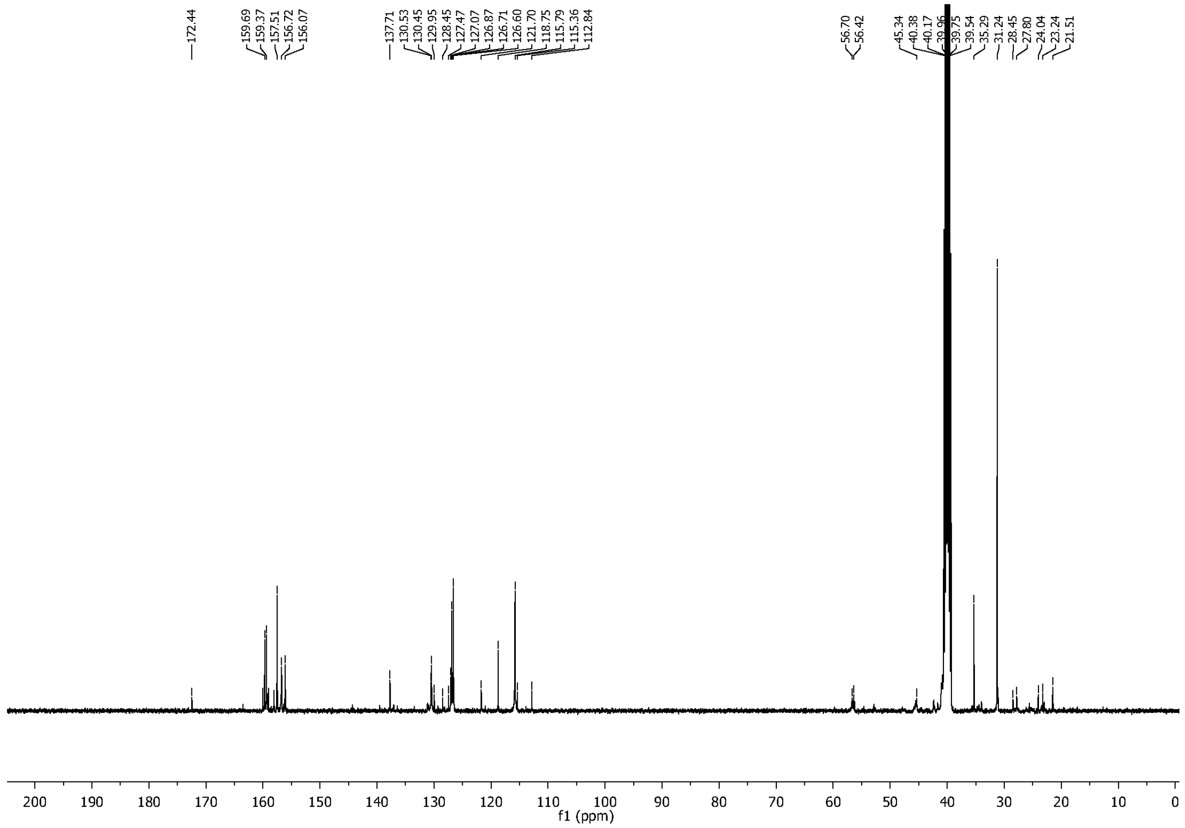
172.44
159.69
159.37
157.51
156.72
156.07
137.71
130.53
130.45
129.95
128.45
127.47
127.07
126.87
126.71
126.60
121.70
118.75
115.79
115.36
112.84
56.70
56.42
45.34
40.38
40.17
39.96
39.75
39.54
35.29
31.24
28.45
27.80
24.04
23.24
21.51
f1 (ppm)
